# Determination of GP88 (progranulin) expression in breast tumor biopsies improves the risk predictive value of the Nottingham Prognostic Index

**DOI:** 10.1186/s13000-016-0520-4

**Published:** 2016-08-08

**Authors:** Ginette Serrero, Douglas M. Hawkins, Pablo A. Bejarano, Olga Ioffe, Katherine R. Tkaczuk, Robert E. Elliott, Jonathan F. Head, Jeffrey Phillips, Andrew K. Godwin, JoEllen Weaver, David Hicks, Binbin Yue

**Affiliations:** 1 A&G Pharmaceutical Inc., Columbia, MD USA; 2 School of Statistics, University of Minnesota, Minneapolis, MN USA; 3 Cleveland Clinic Florida, Weston, FL USA; 4 Program of Oncology, University Maryland Greenebaum Cancer Center, Baltimore, MD USA; 5 Elliot, Elliot, Head Breast Cancer Research & Treatment Center, Baton Rouge, LA USA; 6 University of Kansas Medical Center, Kansas City, KS USA; 7 University of Pennsylvania School of Medicine, Philadelphia, PA USA

**Keywords:** Breast Cancer, Biomarker, GP88, Progranulin, Nottingham Prognostic Index, Prognostic, Immunohistochemistry

## Abstract

**Background:**

The Nottingham Prognostic Index (NPI), which combines numerical values for nodal status, tumor size and histological grade, is used in the standard of care to provide predictive value information on post-surgery survival for patients with primary breast cancer. Attempts to improve the performance of the NPI algorithm have been carried out by testing the inclusion of other biomarker expression and morphological features such as vascular invasion. In the present study, we investigated whether expression of the autocrine growth and survival factor GP88 (progranulin), known to be overexpressed in breast cancer, would improve NPI’s predictive value.

**Methods:**

We examined by immunohistochemistry (IHC) the GP88 expression in 508 cases of estrogen receptor positive invasive ductal carcinoma with known clinical outcomes and for which NPI had been determined. GP88 IHC expression was scored by two board certified pathologists and classified into two score groups of GP88 <3+ (0, 1+, 2+) and GP88 = 3+. The correlation between GP88 scoring, NPI and disease-free (DFS) or overall survival (OS) outcomes was then examined by Kaplan-Meier analysis, Cox proportional Hazard (CPH) ratio and Pearson’s *X*^2^ test.

**Results:**

Kaplan-Meier survival graphs of cases categorized by their NPI scores (<3.4, 3.4–5.4, >5.4) and GP88 expression showed that for patients within the same NPI subgroup, patients having tumors with a high GP88 expression (GP88 IHC score of 3+) had a worse DFS than patients with tumors that had a low GP88 expression (GP88 IHC score <3+). When adjusted for NPI, high GP88 score was significantly associated with recurrence with a hazard ratio of 3.30 (95 % CI 2.12 to 5.14).

**Conclusions:**

The data suggest that the determination of GP88 tumor expression at time of diagnosis for early stage breast cancer patients can provide additional survival information to that provided by NPI alone and thus may be useful for risk management of patients diagnosed with breast cancer.

## Background

Prognostic factors are used to provide information on the clinical management of patients. In the case of breast cancer patients, the common prognostic factors are tumor size, histological grade, histological nodal status and patient’s age. Estrogen and progesterone receptor and HER-2 expression provide additional information and guideline for treatment decisions. Additionally, proliferation markers such as DNA ploidy, S fraction or Ki67 expression are increasingly examined and incorporated for risk evaluation at time of diagnosis. Such factors might be used to discriminate among patients at increased risk of recurrence from the ones at low risk and thus identify patients that may benefit from adjuvant therapy from the ones more likely to display treatment resistance. Some tumor characteristics have been used to define a prognostic index such as the Nottingham prognostic index (NPI) proposed in 1982 [1].

The NPI was initially derived from a retrospective study of 382 patients with operable primary breast cancers. This finding was confirmed after long-term follow-up [2] and was independently validated in other multi-center studies [3, 4]. The calculated NPI predicting survival includes tumor and disease characteristics such as tumor size, histological grade and nodal status and is defined by the following formula: NPI = tumor size (cm) x 0.2 + grade (I-III) + lymph node score (1–3). Patients are typically stratified into three NPI categories associated with different survival outcomes: <3.4 (good prognosis group), 3.4–5.4 (medium prognosis group) and >5.4 (poor prognosis group). As with most prognostic algorithms, within the stratified NPI groups, there are patients that do less well than the rest of the group and as such there is a need to determine if NPI stratification can be further improved to better stratify patients within the NPI groups.

In recent years, there have been several attempts to increase the predictive value of NPI by combining NPI scores with the expression of prognostic biomarkers [5, 6]. In the present paper, we examined the predictive value of the autocrine growth and survival factor GP88/Progranulin in combination with NPI to determine whether adding GP88/Progranulin tumor expression determination to NPI scoring could provide additional prognostic information and further stratify patients in low and high risk recurrence groups within each NPI category.

The 88 kDa, cysteine-rich glycoprotein GP88 (also known as Progranulin, PCDGF, granulin/epithelin precursor or acrogranin) is the largest member of a unique family of growth factors that plays a role as growth and survival factor and is characterized by 7 and a half repeats of a distinct double cysteine-rich granulin/epithelin motif [7–9]. Initially identified as being overexpressed in breast cancer, GP88 has since been reported by many investigators to be overexpressed in several other human cancers while normal corresponding tissues display little or no GP88 expression [10–12].

In breast cancer, GP88 expression is associated with increased tumorigenesis and it mediates in part, cancer cell growth, survival, resistance to therapy (anti-estrogen, Herceptin and doxorubicin) and several hallmarks of metastasis such as invasion, angiogenesis and migration [13–16]. The pathways activated by GP88 signaling include the mitogen-activated protein kinase (ERK 1/2), and phosphatidylinositol 3-kinase (PI-3 K), leading to the activation of the cell cycle regulatory proteins such as Cyclin D1, Cyclin B and CDK4 [14, 17, 18].

In human breast carcinomas, GP88 is highly expressed in both estrogen receptor positive (ER^+^) and estrogen receptor negative (ER-) cells. Inhibition of GP88 expression by GP88 antisense cDNA or SiRNA resulted in inhibition of cell proliferation and reduction of tumor incidence and tumor size in nude mice [14]. In ER^+^ cells, GP88 overexpression was associated with estrogen independence and acquisition of resistance to the anti-estrogen tamoxifen, faslodex and the aromatase inhibitor letrozole [17, 19, 20]. Immunohistochemistry (IHC) studies of formalin-fixed paraffin-embedded (FFPE) tumor specimens demonstrated that GP88 tumor tissue expression was low or negative in normal mammary tissues and lobular carcinoma whereas it was elevated in ductal carcinoma *in situ* (DCIS) and invasive ductal carcinoma (IDC) tissues [21]. In IDC, high GP88 expression positively correlated with p53 expression and Ki67 index whereas it was independent of HER2 expression [21]. Based on the fact that GP88 expression in ER^+^ cells was associated with estrogen independence and tamoxifen resistance [17], analysis of GP88 tissue expression in ~600 cases of ER^+^ IDC in relation with clinical outcomes demonstrated that high GP88 expression (IHC score of 3+) was associated with a 5.9-fold higher hazard of disease recurrence (*p* < 0.0001) and a 2.5-fold higher mortality hazard (*p* = 0.0002) compared to patients with no or low tumor GP88 expression [22]. Since GP88 is a secreted protein, it can also be found in the circulation and is measurable in serum using an Enzyme Immunoassay (EIA) developed in our laboratory. A longitudinal clinical study demonstrated the performance of the serum GP88 EIA by establishing a basal range for GP88 in serum from healthy volunteers of 28.7 ± 5.8 ng/ml and showing that serum GP88 levels in breast cancer patients was elevated to 40.7 ± 16.0 ng/ml in early stage and over 100 ng/ml in later stages of breast cancer [23]. These studies demonstrated the importance of GP88 as a risk predictor of breast cancer survival.

Based on these observations, the present study focused on determining whether associating GP88 IHC tumor tissue scores to NPI determination would increase NPI predictive value and further stratify breast cancer patients for risk.

## Methods

### Study populations

The breast cancer patient cohort used for this study consisted of 574 cases of ER^+^ IDC diagnosed between 1985 and 2003 collected from six geographically distinct US institutions: Kaiser Permanente, (Portland, OR), Kaiser Permanente (Miami, FL), Washington University, (St Louis, MO), University of Miami, (Miami, FL), Fox Chase Cancer Center (Philadelphia, PA), the EEH Breast Cancer Research and Treatment Center (Baton Rouge, LA). The first four sites were part of the Cooperative Breast Cancer Tissue Resources (CBCTR) from the National Cancer Institute [24].

The retrospective patients’ information and material were de-identified and given new unique case numbers prior to shipment. The study was reviewed and approved by the Chesapeake Research Review’s IRB (CRRI 1006001). The board confirmed that informed consent was not required for this study.

Upon histological examination, 31 cases contained slides with no evaluable tumor tissue and 35 additional cases were missing some tumor characteristics information required to determine NPI. As a result, these cases were excluded from the final analysis. Therefore, the final database for analysis included 508 cases. The information about tumor size, lymph node status and tumor grade for each patient provided in the database was used to determine their NPI using the formula: NPI = Size (cm) x 0.2 + grade (1–3) + lymph node score (1–3). Three NPI categories were used: < 3.4 (good prognosis group), 3.4–5.4 (medium prognosis group) and >5.4 (poor prognosis group) to stratify patients for analysis as described in published reports.

### GP88 expression by Immunohistochemistry

GP88 tissue expression was measured by IHC on sections of tissue from FFPE whole tissue blocks using previously validated and described IHC methodology [21, 22]. Briefly, for each case, individual 5 micron sections on positively charged microscope slides were deparaffinized with xylene and rehydrated through a graded ethanol series. Antigen retrieval was conducted for 25 min in 0.2 M citrate buffer pH 6.0 in a 94 °C water bath. Staining was carried out on a Dako Autostainer. GP88 was detected in tissue sections by incubation with an anti-human GP88 mouse monoclonal antibody, clone 6B3 from A&G Pharmaceutical Inc. (Precision Antibody Division) Columbia, MD, followed by washing, and incubation with HRP-conjugated secondary goat anti-mouse antibody (Dako, Carpinteria, CA). Bound antibody was detected using DAB as chromogen (Dako). Slides were then washed and counter-stained with Mayer’s Hematoxylin, prior to examination and scoring.

### Evaluation of GP88 immunohistochemistry results

GP88 cytoplasmic immunoreactivity was semi-quantitatively scored as: <10 % of cells staining: negative (0); >10 % of cells staining: positive with positive staining graded from weak/focal (1+) to moderate/focal or diffuse (2+) to strong/diffuse (3+) as described previously [21, 22]. The immuno-stained slides were evaluated and scored by two board certified pathologists who independently examined the tissue sections while blinded to the clinical data.

### Statistical analysis methods

Descriptive statistics were used to summarize the patients’ and tumors’ characteristics from these 508 cases. Using the data from the 508 ER^+^ IDC cases, we established the disease-free survival (DFS) and overall survival (OS) for each patient. DFS was defined as the time interval from date of diagnosis to first recurrence (local or distant). In the same patient population, OS was defined as the time interval from date of diagnosis to time of last follow-up or death, regardless if breast cancer was the primary or underlying cause of death. Time to recurrence was censored at the time of last disease-free follow-up, and at death for those patients who died without a previous recurrence.

The relevance of the NPI score to survival was verified by fitting Kaplan-Meier (KM) curves for DFS and OS stratified by NPI and the curves were compared using the log rank tests. As an initial exploration of the interplay between NPI and GP88, KM curves stratifying survival by GP88 within each NPI category were fitted. P values of the log rank test for the relevance of GP88 within each NPI category were calculated.

Finally, multivariate analyses using Cox Proportional Hazard (CPH) models were carried out to test whether the two markers (GP88 and NPI) provided separate information, additional to that delivered by other markers for DFS and OS. All the calculations were performed in R [25].

## Results

### Determination of the NPI scores of the study population

508 ER^+^ IDC cases were obtained from 6 geographically distinct US institutions as described in the method section. Cases were de-identified and obtained along with clinical and pathological parameters provided by the tissue repositories. These included age at diagnosis, disease stage, tumor size, tumor grade, steroid receptor status (estrogen and progesterone receptors), lymph node status, adjuvant treatment (chemotherapy, hormone therapy) and clinical outcomes such as recurrence and survival information. All patients underwent surgery and none of the patients received neo-adjuvant therapy. Since the menopausal status of patients was not provided in the database, age was used as a surrogate, with patients aged >50 years old considered to be post-menopausal for this analysis. Estrogen receptor status and progesterone receptor status for the cases examined had been determined by IHC using Ventana IHC kits (Ventana, Tucson, AZ). All cases examined were ER^+^. Median follow-up was 91.3 months. Descriptive statistics were used to present the patient and tumor characteristics (Table 1). NPI score was determined using the formula provided in the method section. The distribution of NPI scores within the 3 NPI categories (GPG ≤3.4; MPG 3.4–5.4; PPG >5.4) provided in Table 1 shows that the patients were fairly evenly distributed among the three NPI categories.

**Table 1:** Patients and tumor characteristics of the study cohort

Characteristics	Groups	Number	Percent
Age at Dx	Median	61	N/A
	Range	24–93	N/A
Race	Caucasian	276	54
	African American	42	8
	Asian	8	2
	Unknown	182	36
ER	Positive	508	100
PR	Positive	307	60
	Negative	140	28
	Unknown	61	12
Tumor size	<2.5 cm	346	68
	2.5–5 cm	137	27
	>5 cm	25	49
Tumor grade	Grade 1	46	9
	Grade 2	220	43
	Grade 3	242	48
Stage	Stage 1	221	44
	Stage 2	231	45
	Stage 3	56	11
Lymph node	Negative	267	53
	Positive	241	47
NPI	≤3.4	155	30
	3.4–5.4	244	48
	>5.4	109	22
GP88	<3+	446	88
	3+	62	12

**Fig. 1:**
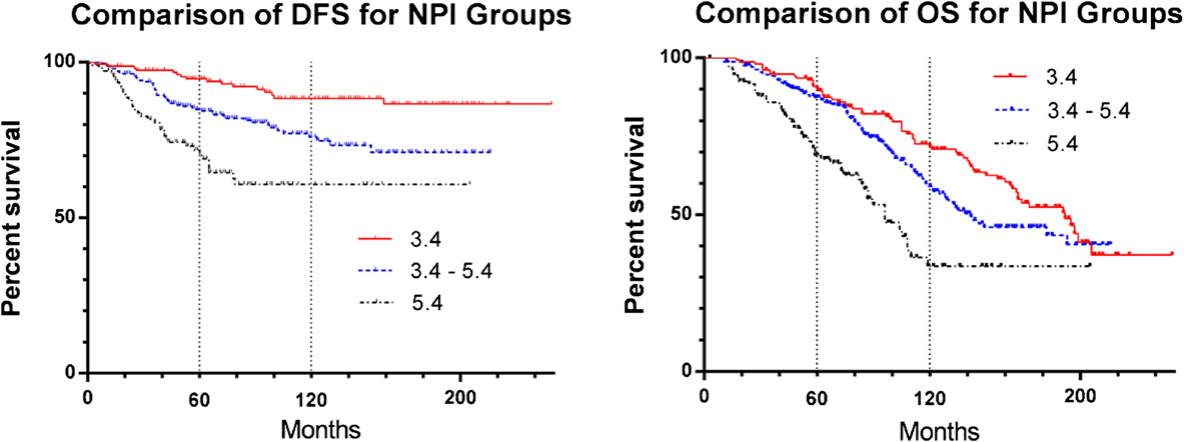
Disease Free Survival and Overall Survival for patients within each NPI category. The NPI was calculated for each of the 508 ER^+^ IDC cases. Kaplan-Meier plots were fitted for DFS and OS for each of the 3 NPI categories (GPG <3.4 in red, MPG 3.4–5.4 in blue, GPG >5.4 in *black*)

A Kaplan-Meier analysis of DFS and OS for each of the 3 NPI categories showed that as expected, DFS and OS decreased when NPI score increased (Fig. 1). This confirmed the relevance of the NPI in DFS and OS for this patient population. Table 2 shows a cross-tabulation of the patients by NPI category and identifies the DFS probability at 60 months and 120 months for patients in each NPI category. The table also lists the 95 % confidence interval for the difference in survival probability between each NPI category and the category above it, at each of these times. The data show that DFS and OS probabilities for the study population are within accepted ranges for the published NPI categories based on published reports and confirm that as NPI score increases, the patient survival decreases.

**Table 2:** Distribution of DFS in the patient population in each NPI category

NPI Score	Percent disease-free survival	
	60 months	120 months
≤3.4	94.6 (1.9) %	88.3 (2.9) %
3.4 to 5.4	84.3 (2.4) %	76.0 (3.3) %
CI	4.2–16.4 %	3.6–21.0 %
>5.4	70.4 (4.7) %	60.8 (5.5) %
CI	3.3–24.2 %	2.4–28.0 %

The percent disease-free survival (DFS) probability for each NPI group was calculated for 60 and 120 months, standard errors are included in parenthesis. The 95 % Confidence Intervals (CI) for the difference in survival probability for ≤3.4 vs 3.4 to 5.4 and 3.4 to 5.4 vs >5.4 for each time-point are listed

### Stratification of NPI categories by GP88 expression

The 508 patients that were stratified in the three NPI categories had their IHC GP88 tissue expression determined and scored as described in the method section. GP88 IHC scores were then grouped as IHC scores of 3+ and <3+ (0, 1+, 2+) as proposed previously [21, 22]. Representative photomicrographs of the staining for all GP88 scores have been previously published [22]. Overall 12.2 % of the cases examined had elevated GP88 expression levels. A cross-tabulation of NPI score against GP88 (Table 3) showed a strong association between NPI and GP88 (chi-squared = 15.28, *P* = 0.0005). Going from the lowest NPI risk category to the highest, the proportion of patients with elevated GP88 expression increased from 5.2 to 21.1 %, in agreement with the fact that higher NPI category corresponded to decreased survival probability and that increased GP88 expression was also associated with worse outcome.

**Table 3:** Distribution of GP88 expression within each NPI category

NPI	GP88		
	< 3+	3+	Total
≤3.4	147 (94.8 %)	8 (5.2 %)	155 (30.5 %)
3.41–5.4	213 (87.3 %)	31 (12.7 %)	244 (48.0 %)
>5.4	86 (78.9 %)	23 (21.1 %)	109 (21.5 %)
Total	446 (87.8 %)	62 (12.2 %)	508 (100 %)

We have previously established that GP88 expression was strongly related to OS and to DFS [22]. The data of Table 3 raised the question of whether the prognostic value of GP88 was a consequence of this association with conventional risk measures as summarized by NPI or whether GP88 carried separate or additional prognostic information on its own.

### GP88 tissue expression further stratifies DFS and OS with each NPI category

**Fig. 2:**
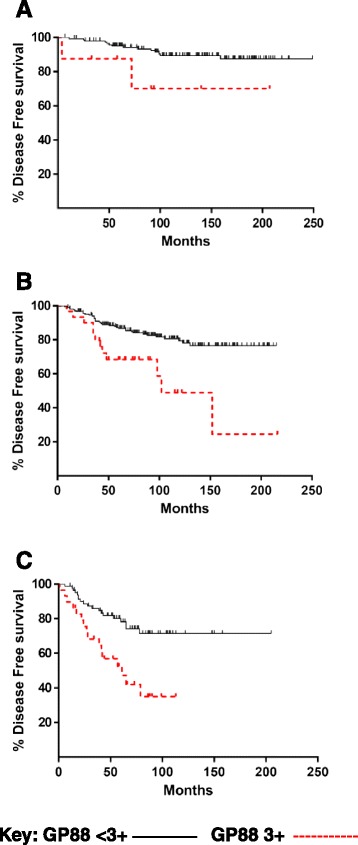
Disease Free Survival by GP88 score within the three NPI groups. Using the NPI score calculated for each of the 508 ER^+^ IDC cases, Kaplan-Meier plots were fitted for DFS functions for the patients within each NPI grouping (**a**-GPG <3.4, **b**-MPG 3.4–5.4, **c**-GPG >5.4) stratified by their GP88 tissue expression using the IHC scores of <3+ in black solid line and 3+ in red dashed line

**Fig. 3:**
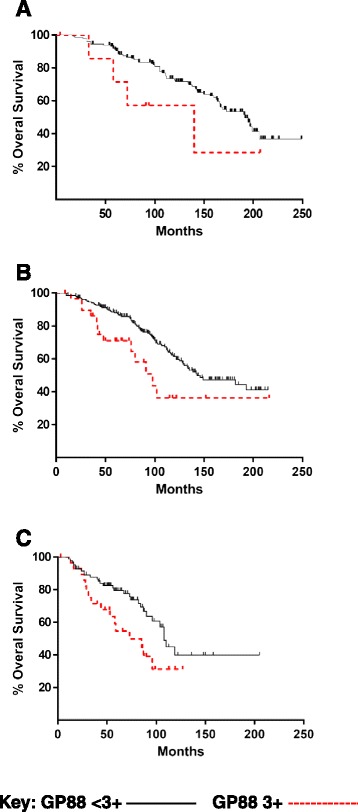
Overall Survival by GP88 score within the three NPI groups. Using the NPI score calculated for each of the 508 ER^+^ IDC cases, Kaplan-Meier plots were fitted for OS functions for the patients within each NPI grouping (**a**-GPG <3.4, **b**-MPG 3.4–5.4, **c**-GPG >5.4) stratified by their GP88 tissue expression using the IHC scores of <3+ in black solid line and 3+ in red dashed line

GP88 prognostic information was investigated in two analyses. In the first, we examined Kaplan-Meier DFS and OS functions for the patients within each NPI grouping separated by their GP88 tissue expression with IHC scores of <3+ and 3+ (Figs. 2 and 3). The data showed that within each NPI category, the DFS (Fig. 2 a–c) and OS (Fig. 3 a–c) probabilities of patients with elevated GP88 (IHC score 3+) were lower than that of patients with lower GP88 expression levels (IHC score <3+). High GP88 expression was associated with worse survival in each of the three NPI categories in Figs. 2 and 3 and this was quantified by the significant p values of the associated logrank statistics as shown in Table 4. This table shows high significance for GP88 in DFS in the NPI 3.4–5.4 and >5.4 groups (*p* = 0.0002 and *p* = 0.0005 respectively), but modest statistical significance in the DFS NPI <3.4 group (*p* = 0.0698). In comparison, GP88 showed statistical significance in the >5.4 NPI OS group (*p* = 0.0343) while in the other two groups GP88 showed modest or no significance. However, this conclusion is limited due to the small number of OS events in these comparisons.

**Table 4:** Significance of GP88 within NPI groupings

NPI	DFS	OS
≤3.4	0.0698	0.2331
3.4 to 5.4	0.0002	0.0628
>5.4	0.0005	0.0343

The final analysis performed was to quantify the information from the KM analysis in a simultaneous test. This second analysis was done using the CPH model with DFS and OS as dependent variables. Three covariates were used: the elevated GP88 indicator, an indicator of NPI >3.4 and an indicator for NPI >5.4.

Table 5 demonstrates that, when adjusted for NPI, elevated GP88 was highly significantly associated with recurrence. Its hazard ratio was 3.30 (95 % CI 2.12 to 5.14). Having an NPI between 3.4 and 5.4 rather than ≤3.4 was also highly significant. It corresponded to a hazard ratio of 2.13 (95 % CI 1.21 to 3.76). If the NPI exceeds 5.4, this added a further highly significant hazard for recurrence – HR = 1.90 (95 % CI 1.23 to 2.95).

**Table 5:** CPH model for DFS

Variable	Hazard Estimate	SE	Chi-square	P > ChiSq	Ratio
GP88 = 3+	1.19337	0.22639	27.7862	<.0001	3.298
NPI >3.4	0.75622	0.29057	6.7733	0.0093	2.130
NPI >5.4	0.64347	0.22371	8.2738	0.0040	1.903

Concerning OS, Table 6 shows that, when adjusted for NPI, GP88 was a highly significant indicator of overall mortality with a hazard ratio of 1.89 (95 % CI 1.28 to 2.80). An NPI between 3.4 and 5.4 was not significantly worse than NPI ≤3.4 but an NPI >5.4 was also a highly significant indicator of mortality, with a hazard ratio of 1.95 (95 % CI 1.38 to 2.77).

**Table 6:** CPH model for OS

Variable	Hazard Estimate	SE	Chi-square	P > ChiSq	Ratio
GP88 = 3+	0.63731	0.20018	10.1362	0.0015	1.891
NPI >3.4	0.24870	0.16564	2.2544	0.1332	1.282
NPI >5.4	0.66977	0.17789	14.1766	0.0002	1.954

## Discussion

The ability to accurately evaluate risk of recurrence remains a challenge in the current standard of care for breast cancer patient management. In recent years molecular biology based tests such as Oncotype Dx (Genomic Health, Redwood City, CA) and MammaPrint (Agendia, Irvine, CA) have been developed for stratifying certain populations of breast cancer patients for risk of recurrence based on the profile of several target genes [26, 27]. However, such tests remain expensive and the results are not applicable to all breast cancer populations. Alternatively, the use of a combination of markers such as ER, PR, HER-2 and Ki67 together with disease characteristics such as tumor size and lymph node involvment has been somewhat useful in providing a risk of recurrence assessment for breast cancer patients at primary diagnosis. Since ER, PR, HER-2 and Ki67 are all cost effective laboratory tests that can be performed in most pathology laboratories, the investigation of additional protein-based tissue biomarkers that are useful in risk of recurrence prediction is important to improve the clinical management of breast cancer patients.

The NPI is a widely used prognostic index based on a combination of histopathological features which are strong independent predictors of clinical outcomes such as lymph node status, tumor size and tumor grade for patients with invasive ductal carcinoma. NPI scores stratify patients into three prognostic categories: good prognosis group (GPG) with an NPI score of ≤3.4; medium prognostic group (MPG) with an NPI score 3.4–5.4; and poor prognosis group (PPG) with an NPI score of >5.4. Patients in the GPG group are potentially spared chemotherapy and the associated side effects. In support for its applicability to breast cancer patient management, NPI has been verified prospectively and validated in two large multicenter studies involving close to 11,000 patients [3, 4].

One of the great advantages of NPI is its simplicity. However, diverse strategies to improve the predictive value of NPI have been explored. In particular, combining the analysis of certain biomarkers with NPI determination has resulted in providing additional risk prediction information and refining the value of the NPI determination. Multiple biomarkers of relevance to the biology of mammary tumors have been investigated by several laboratories. Callagy et al. [5] analyzed the expression of 13 biomarkers. They concluded that in univariate analysis eight biomarkers showed a significant association with survival at 10 years. Out of these 8 biomarkers, only bcl-2 retained prognostic significance independent of NPI. They concluded that measuring bcl-2 expression in tumor biopsies was an independent predictor of breast cancer outcomes and could be useful as a prognostic adjunct to NPI, particularly in the first 5 years after diagnosis. Parisi et al. [28] outlined the benefits of the inclusion of biomarkers with clinico-pathological covariate in breast prognostic models. They examined the expression of 14 biomarkers out of the 21 present in the Oncotype Dx test (Genomic Health) in addition to tumor characteristics found in NPI. They showed that in lymph node negative ER^+^ tumors, three biomarkers Aurora Kinase 1, CD68 and HER-2 provided additional predictive information. The inclusion of other factors such as vascular invasion, basal phenotype and HER-2 status has also been considered. Of these factors, the one with the most evidence to support its inclusion is vascular invasion in patients with node-negative disease [29, 30].

Since the survival factor GP88 (progranulin) is preferentially expressed in invasive ductal carcinoma and plays a role in breast cancer cell aggressiveness, we have hypothesized that measuring GP88 expression in tumor tissues can also provide additional risk prediction information and increase the value of NPI determination. We have shown previously that GP88 expression was associated with decreased DFS for patients with ER^+^ IDC [22]. We show here that determination of GP88 tumor tissue expression further stratifies ER^+^ IDC patients by their DFS within each NPI category. This would suggest that GP88 provides additional information to that provided by NPI alone and thus can be useful for risk management of the patients. It is interesting to note that the NPI >5.4 group of patients with a low GP88 expression (Fig. 2c) had DFS outcome similar to the low NPI category with high GP88 expression (Fig. 2a). This finding would suggest that even for the GPG outcome group (NPI <3.4), the fact of having a tumor GP88 expression of 3+ brings this subset of patients to a similar DFS as the PPG outcome group (NPI >5.4). This would suggest that a combination of low NPI score and low GP88 score is required to produce a favorable DFS outcome. Additionally, no matter which NPI group the patient is stratified into, having a low GP88 score contributes to a better DFS outcome.

We do not know whether GP88 expression would have the same impact as an independent risk factor for patients that have ER negative breast tumors. The fact that GP88 expression down regulates ER expression and drives ER^+^ breast cell lines to become estrogen independent would suggest that GP88 could also be a useful prognostic factor in conjunction with NPI for patients with ER^−^ tumors [17]. However, this possibility needs to be directly investigated pending the availability of suitable ER^−^ cases with proper clinical outcomes.

Based on the results described here, the present study provides supportive evidence that routine GP88 determination can be used in the clinic as a complement to NPI stratification to improve risk prognostication for an individual patient, particularly during the first 5 years post- diagnosis of invasive breast cancer. GP88 expression can be measured by IHC in a fast, reproducible and cost effective way. The fact that GP88 determination can enhance the predictive value of NPI would indicate the usefulness of GP88 IHC test along with other biomarkers measured as per the standard of care such ER, PR, HER-2 and Ki67.

Tumor expression of the proliferation antigen Ki67 is currently used to assess the prognosis of cancer patients [31]. In addition, recently, prognostic value of Ki67 expression was demonstrated after short-term pre-surgical endocrine therapy for primary breast cancer [32]. It is interesting to note that GP88 is a growth factor shown to upregulate proliferation markers and as such could complement the information provided by measuring Ki67 expression. In support of this possibility, we have reported previously that high GP88 expression in IDC positively correlated with Ki67 index [21]. A small retrospective study of 85 cases of breast cancer patients with ER^+^ IDC further demonstrated a correlation between GP88 and Ki-67 (*p* < 0.004). In this study, it was interesting to note that the combined GP88 and Ki-67 scores were statistically associated (*p* = <0.03) with OncoType Dx® recurrence score [33]. Retrospective studies with larger number of cases in the lower NPI category and most importantly, prospective studies that include GP88 expression as part of a panel of prognostic and predictive markers will be useful to further validate GP88 diagnostic utility.

## Conclusion

The data suggest that the determination of GP88 tumor expression at time of diagnosis for early stage breast cancer patients could provide additional survival information to that provided by NPI alone and thus may be useful for risk management of patients diagnosed with breast cancer.

## Abbreviations

CBCTR, Cooperative Breast Cancer Tissue Resources; CPH, Cox proportional hazard; DFS, disease-free survival; EIA, Enzyme Immunoassay; ER-, estrogen receptor negative; ER^+^, estrogen receptor positive; FFPE, formalin-fixed paraffin-embedded; GPG, NPI Good prognosis group; IDC, invasive ductal carcinoma; IHC, immunohistochemistry; KM, Kaplan-Meier; MPG, NPI Medium prognostic group; NPI, Nottingham prognostic index; OS, overall survival; PPG, NPI Poor prognosis group.
